# A case report of pulmonary thromboembolism following allergic bronchopulmonary aspergillosis

**DOI:** 10.1097/MD.0000000000018692

**Published:** 2020-01-10

**Authors:** Ying Pan, Feng Xu, Wei Ou-yang

**Affiliations:** Department of Infectious Diseases, Second Affiliated Hospital, Zhejiang University School of Medicine, Hangzhou, China.

**Keywords:** allergic bronchopulmonary aspergillosis, aspergillus, pulmonary thromboembolism

## Abstract

**Rationale:**

Allergic bronchopulmonary aspergillosis (ABPA) complicated with pulmonary thromboembolism (PTE) is rare. This report describes a patient who was diagnosed with ABPA and soon developed PTE.

**Patient concerns:**

A 64-year-old man was diagnosed with ABPA in hospital for recurrent fever with cough. Two months later, the patient was readmitted to the hospital because of PTE.

**Diagnoses:**

ABPA was diagnosed during the first hospitalization, and laboratory tests showed an increase in serum IgE and *Aspergillus fumigatus*-specific IgG. Sputum culture suggested *A. fumigatus* and high-resolution computed tomography (HRCT) showed inflammation of both lungs and central bronchiectasis. During the second hospitalization, the patient's chest angiography showed PTE.

**Interventions:**

The patient began treatment with antifungal drugs and corticosteroids, and was discharged from the hospital when his condition improved. Two months after discharge, the patient was treated with anticoagulant drugs due to PTE.

**Outcomes:**

The patient got better after taking anticoagulant drugs and was discharged from the hospital. The patient appears for regular follow-up visits in our outpatient clinic every 2 months and is currently in good condition.

**Lessons:**

Patients with ABPA may be concurred with PTE. The risk of PTE in ABPA should be assessed in advance and preventive strategies also need to be taken beforehand. Pulmonary artery examination is necessary once it happened.

## 1 Introduction

*Aspergillus* is a ubiquitous fungus isolated from both outdoor and indoor environments. Only a minority of the population consequently develops pulmonary diseases because of the inhalation of *Aspergillus* spores. Depending on the interaction between the fungal burden and host's immune status or immune hyperactivity, pulmonary aspergillosis has a wide spectrum of disease presentations, including chronic pulmonary aspergillosis such as aspergilloma and chronic necrotizing aspergillosis, invasive pulmonary aspergillosis, and allergic bronchopulmonary aspergillosis (ABPA).^[1]^ ABPA is an immunological pulmonary disorder caused by hypersensitivity to *Aspergillus fumigatus*, manifesting as poorly controlled asthma, recurrent pulmonary infiltrates, and bronchiectasis.^[2]^ In the recent years, ABPA has become more and more common clinically, especially in patients with cystic fibrosis or asthma, which can lead to irreversible bronchiectasis, pulmonary fibrosis, and even death. The common complications of ABPA include recurrent exacerbations, bronchiectasis, and acute respiratory failure.^[3]^ It is generally believed that, however, pulmonary thromboembolism (PTE) is a rare complication of ABPA. To our knowledge, no adult case of PTE due to ABPA has been reported. We describe here an elderly man with ABPA who was admitted to hospital because of PTE after regular treatment for 2 months. Fortunately, he recovered well after timely treatment.

## 2 Case report

**Figure 1:**
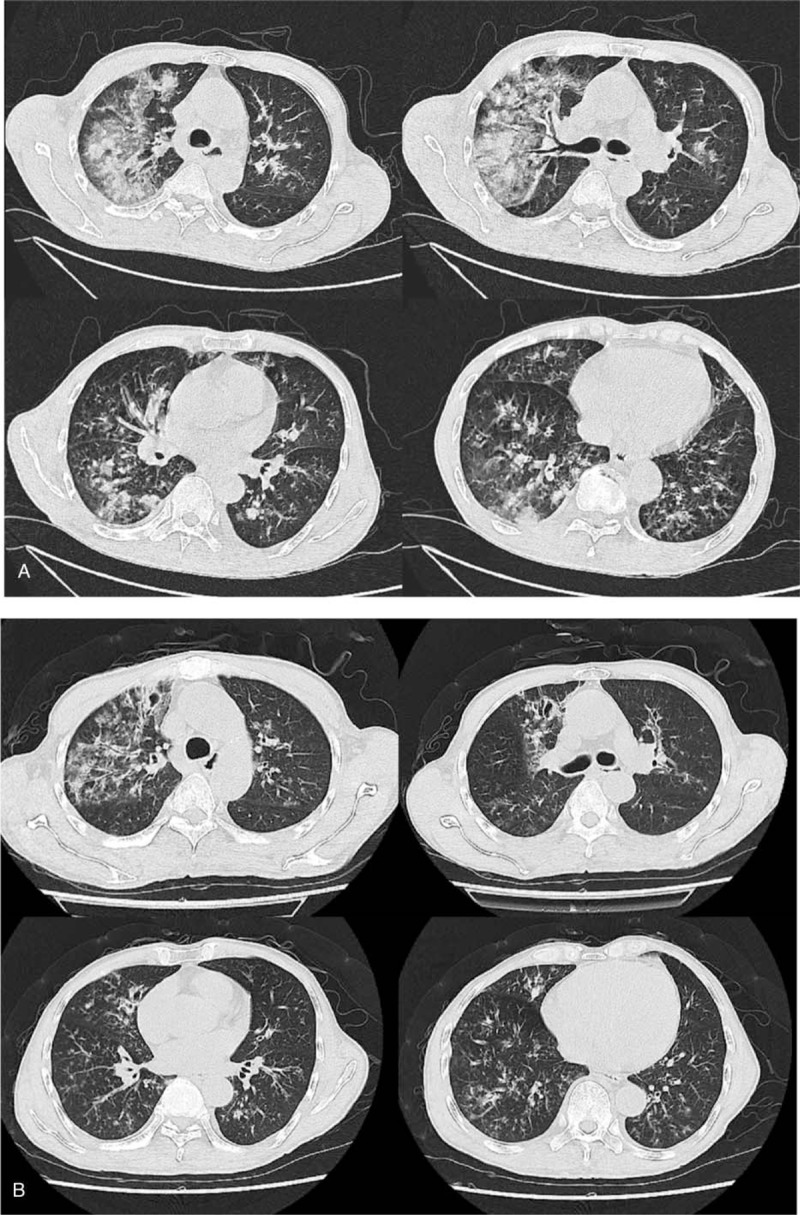
A. HRCT of the chest showing inflammation in both lungs with central bronchiectasis; B. HRCT of the chest showing improved inflammation of both lungs after antifungal treatment. HRCT = high-resolution computed tomography.

A 64-year-old man was hospitalized for “recurrent fever and cough with brown sputum for 7 days.” No tuberculosis or asthma was reported in the family history. The patient had a 20-year smoking history with 30 cigarettes per day. Oxygen saturation was 93% under a 5 L/min oxygen flow rate. Auscultation showed a widespread audible expiratory wheeze on both upper lungs and moist rales on both lower lungs. Breathing sound was low and the patient had the symptom of expiratory dyspnea. Initial laboratory findings showed serum IgE levels of 128.0 U/mL (ULN 100.0) and positive *A. fumigatus* IgG. Sputum culture was performed and *A. fumigatus* grew. Multiple plaque shadows were detected on both lungs by a chest radiograph. Lung inflammation with central bronchiectasis was identified by high-resolution computed tomography (HRCT) (Fig. 1A).

The patient was diagnosed with ABPA and had received regular treatment comprising intravenous methylprednisolone (40 mg/d) and voriconazole (200 mg every 12 hours). After 5 days of treatment, symptoms were significantly improved and the methylprednisolone was administered orally. The HRCT was reviewed and showed that infiltration had been significantly improved (Fig. 1B). Eventually, the patient was discharged with oral steroids and voriconazole (200 mg every 12 hours) treatment.

**Figure 2:**
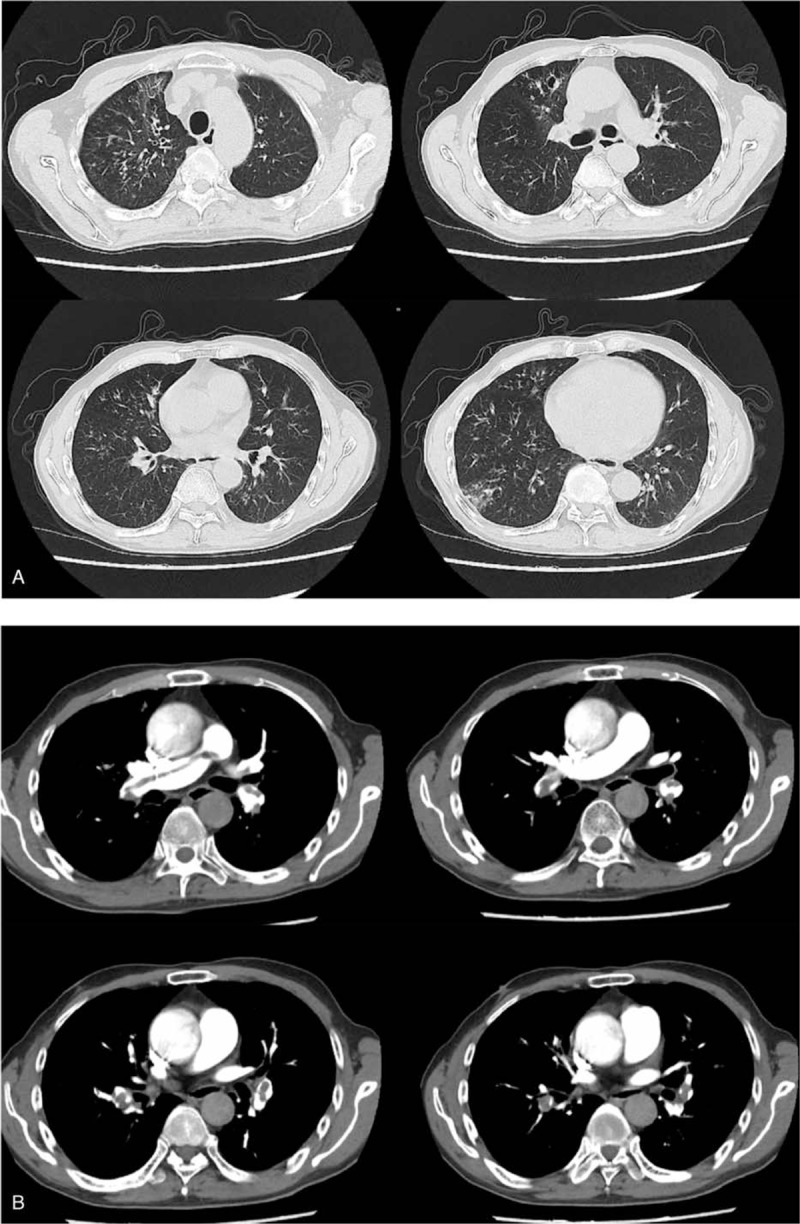
A. HRCT of the chest showing pulmonary infiltrates; B. Chest angiography showing filling defects in the main pulmonary artery and both branches of the pulmonary artery. HRCT = high-resolution computed tomography.

The patient was re-hospitalized for hemoptysis and dyspnea 2 months after discharge. Auscultation found a little moist rale at the base of both lungs. The rest of the physical examination was normal. The result of the D-dimer test was 5630 μg/L (ULN 500 μg/L). HRCT of the chest showed pulmonary inflammation, which indicated an improvement compared with the previous examination (Fig. 2A). However, chest angiography revealed filling defects in the main pulmonary artery and both branches of the pulmonary artery, indicating the occurrence of PTE (Fig. 2B).

The patient was then diagnosed with PTE and received treatment of low molecular heparin (0.4 mL every 12 hours for 7 days) and rivaroxaban (15 mg twice a day). Symptoms gradually improved and the patient was discharged with continued anticoagulant treatment. Currently (over 1 years after diagnosis) the patient is in remission of the disease, and no further thromboembolic complications have been observed.

## 3 Discussion

In this case, the patient met the diagnostic criteria of ABPA, including serum IgE >100 U/L, elevated *A. fumigatus*-specific IgG, HRCT infiltrates, central bronchiectasis, and *A. fumigatus* cultured from the sputum.^[4]^ ABPA is a pulmonary disorder caused by hypersensitivity to *A. fumigatus*. *Aspergillus* species are ubiquitous saprophytes that are widely distributed in nature.^[5]^*Aspergillus* species are usually acquired through inhalation of *Aspergillus* spores in the air. They can commonly lead to life-threatening infections in the sino-nasal region and the upper and lower respiratory tract. In a genetically predisposed individual, inhaled conidia of *A. fumigatus* germinate into hyphae with the release of antigens that activate innate and adaptive immune responses (Th2 CD_4_^+^ T cell responses) in the lungs.^[6]^

ABPA presents varied clinical and radiological manifestations. Patients often present with a productive cough, wheezing, poorly controlled asthma, and hemoptysis. The complications associated with ABPA include recurrent exacerbations, acute respiratory failure due to proximal collapse, and bronchiectasis.^[3]^ However, it's rarely reported that ABPA is associated with PTE. As is known to all, PTE is a disease caused by thrombus blocking of the pulmonary artery or its branches from the venous system or right heart. The main manifestations of PTE are circulatory and respiratory dysfunction, including dyspnea, chest pain, and hemoptysis. There are many risk factors for thrombosis, including age, surgery, brakes, trauma, pregnancy, oral contraceptives, systemic infection, central venous catheterization, obesity, cancer, and lupus.^[7]^

Here, we report a case of ABPA complicated with PTE. As far as we know, adult cases with pulmonary embolism as complication of ABPA have not been reported in the literature. Instead, cases of invasive pulmonary aspergillosis causing infarction with pulmonary necrosis and pulmonary embolism were relatively frequent.^[8–11]^ The underlying mechanisms of ABPA complicated by PTE remain unknown. ABPA can activate the innate immune system of the lung, leading to the increase of several inflammatory cytokines,^[12,13]^ which enter the blood circulation and activate coagulation factor VII. This triggers the extrinsic coagulation pathway and promotes coagulation and thrombosis. In addition, *Aspergillus* can release certain toxic *Aspergillus* proteases.^[14]^ Toxic proteases, chronic hypoxia, and acidosis may directly damage vascular endothelial cells, and thus causing activation of the intrinsic coagulation pathway. Chronic hypoxia also leads to increased levels of compensatory erythrocytes and increased blood viscosity. The decline of liver and kidney function (due to hypoxia and acidosis) affects the clearance of coagulation factors, and it further exacerbates thrombosis accordingly. Therefore, toxins from *Aspergillus* and persistent inflammation may contribute to the pathogenesis of PTE following ABPA.

Since PTE is a life-threatening disorder, clinicians should consider PTE in patients with ABPA, especially in patients who suddenly had symptoms such as dyspnea or ineffective use of antifungal and hormonal drugs. It is important to assess the additional risk of PTE in ABPA patients, and patients at high-risk of PTE should receive prophylactic treatment, unless they have contraindications.^[7]^

In summary, ABPA is a chronic disease with a relapsing remitting course, and the prognosis can be improved by early diagnosis and treatment. ABPA complicated with PTE is extremely rare. Consequently, much more attention should be paid to the ABPA patients with the associated risk factors and/or those who are not responsive to antifungal treatment in consideration of the life-threatening severity of PTE.

## Author contributions

**Conceptualization:** Ying Pan, Feng Xu.

**Data curation:** Ying Pan, Wei Ou-yang.

**Formal analysis:** Ying Pan, Wei Ou-yang.

**Investigation:** Ying Pan, Feng Xu, Wei Ou-yang.

**Methodology:** Ying Pan.

**Resources:** Ying Pan.

**Supervision:** Feng Xu.

**Validation:** Feng Xu.

**Visualization:** Feng Xu.

**Writing – original draft:** Ying Pan.

**Writing – review & editing:** Feng Xu, Wei Ou-yang, Ying Pan.
